# The mediating role of vascular age in the association between blood metals and atherosclerosis from Manganese-exposed workers healthy cohort

**DOI:** 10.1186/s12889-026-26235-5

**Published:** 2026-01-16

**Authors:** Xiaoting Ge, Ying Yang, Junxiu He, Sencai Lin, Yu Bao, Hong Cheng, Haiqing Cai, Fei Wang, Xiaobo Yang

**Affiliations:** 1 School of Medicine, Guangxi University of Science and Technology, Liuzhou, Guangxi, 545006 China; 2 School of Public Health, Guangxi Medical University, Nanning, Guangxi 530021 China

**Keywords:** Metal mixtures, Lead, Atherosclerosis, Vascular age, Mediation

## Abstract

**Background:**

The occurrence and development of atherosclerosis are fundamentally linked to the aging of blood vessels. Previous researches have found that exposure to metals in the environment is linked to atherosclerosis, yet the underlying biological mechanism remains unclear.

**Methods:**

Twelve blood metals, vascular age and brachial-ankle pulse wave velocity (baPWV) were quantified among the 431 individuals involved in Manganese-exposed workers healthy cohort in 2023.

**Results:**

The generalized linear model (GLM) indicated that chromium (Cr) was negatively associated with baPWV (β = -0.041). The GLM, least absolute shrinkage and selection operator and weighted quantile sum (WQS) analysis indicated that lead (Pb) was positively associated with baPWV. Pb contributed the most to the positive association between metal mixtures (Pb, selenium, manganese, Cr, calcium) and baPWV, showing that for every unit increase in the WQS index of metal mixtures, baPWV increased by 0.014 m/s. Subsequently, positive associations were found between Pb and vascular age as well as between vascular age and baPWV. Mediation analysis revealed that vascular age partially mediated (42%, *P* < 0.001) the association between Pb and baPWV. Additionally, joint effect analyses revealed that smoking, drinking, older age and higher BMI might enhance the association between Pb exposure and baPWV.

**Conclusions:**

Vascular age can partly mediate the association between Pb exposure and baPWV, showing higher Pb level related to higher atherosclerosis risk. And healthy habits and BMI may help mitigate the harmful effects of Pb. Further prospective and mechanistic studies are recommended to corroborate the findings and clarify the elusive mechanism.

**Supplementary Information:**

The online version contains supplementary material available at 10.1186/s12889-026-26235-5.

## Introduction

Globally, cardiovascular disease (CVD) significantly contributes to death rates and incurs substantial healthcare expenses. Cardiovascular-related deaths are projected to increase from 20.5 million in 2025 to 35.6 million by 2050 [1], and the rough DALYs will increase by 54.7%. Arterial stiffness serves as a powerful predictor of cardiovascular events and mortality [2, 3]. Therefore, controlling arteriosclerosis is a key link in the prevention and control of CVD. Arterial stiffness is an early sign of changes in the vessel wall’s structure and function, and is characterized by thickening of the inner lining of the arteries, increasing stiffness of the vessel walls, and reducing elasticity [4]. Despite various prevention strategies and treatment advancements, harmful health behaviors and environmental factors have undermined these achievements in CVD control.

Metals, a prevalent category of environmental chemicals, continue to pose a public health risk and are recognized as risks associated with arterial stiffness [5]. Epidemiological researches have also revealed that metals may influence arterial stiffness, leading to CVD. For instance, a cross-sectional investigation in rural India demonstrated that increased levels of arsenic (As) and zinc (Zn) in urine were associated with a higher risk of atherosclerosis [6]. In addition, a panel study based on Chinese community residents has provided evidence of the relationship of exposure to titanium (Ti) and cobalt (Co) with atherosclerosis [7]. Moreover, the evidence from the American Heart Association indicated that exposure to low level of lead (Pb), cadmium (Cd) and As was associated with vascular injury and atherosclerotic diseases [8]. People working in certain environments frequently encounter complex metal mixtures, however, there are few studies assessing the relationship of metal exposure with arteriosclerosis in these populations. According to a cross-sectional study, occupational exposure to lead was associated with significantly higher arterial stiffness in workers compared to healthy individuals [9]. A total number of 25 participants from a cross-sectional study reported that cumulative exposure to nickel (Ni) was associated with the arterial stiffness of welders [10]. However, previous studies in occupational populations have focused mainly on single metal exposure, failing to reflect the true situation of metal mixture exposure, and may underestimate the cumulative impact of metal mixtures on atherosclerosis.

Metals can cause arterial stiffness by triggering oxidative stress and impairing endothelial function [11–13], or by affecting other risk factors for CVD such as diabetes and obesity. However, the biological mechanisms that might explain the link between metal exposure and arteriosclerosis are not yet fully understood. Vascular aging is considered as a crucial contributor to onset and advancement of atherosclerosis [14]. Vascular age is a reliable measure for vascular aging, which helps in the early recognition of individuals with disproportionately high cardiovascular risks [15]. Vascular age has been proven to be highly sensitive to environmental metal exposure [8, 16, 17]. A prevailing hypothesis suggests that metal can trigger oxidative stress and inflammation responses, resulting in changes in both the structure and function of blood vessels and promoting vascular aging [18]. Nevertheless, studies investigating whether vascular age mediates the relationship between metal exposure and atherosclerosis are still scarce.

Since vascular age is associated with heavy metal exposure and atherosclerosis, it is of interest to explore whether vascular age mediates the association between metal exposure and atherosclerosis. However, no previous study has addressed this question. Since humans are susceptible to co-exposure to metals, determining impact of simultaneous metal exposure on vascular aging and atherosclerosis is also of interest. A cross-sectional study was executed among the occupational population from Guangxi, China, for evaluating the relationship of exposure to metal mixtures with arteriosclerosis, and exploring a potential mediating role of vascular age.

## Materials and methods

### Study population

This cross-sectional study was on the basis of the follow-up of manganese-exposed workers healthy cohort (MEWHC). More specifics about the MEWHC can be found elsewhere [19, 20]. Briefly, participants who were aged 18–60 years, worked at the ferro-manganese refinery for at least one year and resided in the local area were enrolled. In 2023 run follow-up, participants completed a structured questionnaire, physical evaluations and measurements and brachial-ankle pulse wave velocity (baPWV) measurement were enrolled (n = 450). Individuals with missing metal concentration data (n = 14) and those diagnosed with cardiovascular diseases or malignant tumors (n = 5) were excluded. Finally, 431 participants were included for the present analysis.

This research received approval from approved by the Ethics and Human Committee of Guangxi Medical University. Before collecting samples and conducting interviews, each participant provided written informed consent.

### Quantifying metals in blood samples

The concentrations of 12 metals in blood, including calcium (Ca), titanium (Ti), chromium (Cr), manganese (Mn), iron (Fe), cobalt (Co), copper (Cu), zinc (Zn), arsenic (As), selenium (Se), cadmium (Cd) and lead (Pb) were measured via inductively coupled plasma mass spectrometry (Thermo Fisher Scientific, USA). The choice of 12 metals was informed by past studies reporting their associations with atherosclerosis. The detection process was conducted following a previously validated and reliable analytical method [21]. In brief, each 100 μL blood sample underwent a 20-fold dilution with a matrix-matching solution composed of 0.1% HNO_3_, 0.5% n-butanol, and 0.1% Triton™ X-100. For quality control, certified reference materials (Seronorm™ Trace Elements Whole Blood RUO no. 210105, 210,205, and 210,305, ALS Scandinavia, Sweden) along with a standard reference material (SRM1640a, Trace Elements in Natural Water from the Natural Institute of Standard Technology, Gaithersburg, MD, USA) were utilized. Parallel analyses were conducted for every 25 samples. The recovery of the internal standard ranged from 70% to 130%. For the 12 blood metals, the detection limits (LOD) varied between 0.001 μg/L and 1.933 μg/L. The detection rate for all metals except Cr was 100% (Table S1), and metal concentrations below the LOD were replaced by the LOD/√2. The results of quality control are shown in Table S2. Since there are no certified values for the metals Ti, Fe and Rb, the spiked recovery test was used to assess the quality of the assay. The results showed that the average values of spiked recoveries for Ti and Fe were 85.92%-124.00% and 84.74%-119.12%, respectively.

### Measurement of baPWV

Measurement of baPWV was conducted using the device for arterial stiffness detection (BP-203RPEIII; Omron, Kyoto, Japan) in accordance with standard protocols. BaPWV was measured by trained technician who had undergone standardized training. The participants were instructed to refrain from smoking, drinking, and ingesting caffeinated beverages for at least one hour prior to the examination. Before the measurement, participants were required to rest for a minimum of five minutes, and all assessments were performed in the supine position. To calculate the baPWV value, the distance between the brachium and the ankle was recorded and further divided by the time taken for the pulse wave to transmit. Higher values of the left and right baPWV were considered in the final analysis.

### Assessment of vascular age

The calculation of vascular age was conducted adhere to the Framingham risk score (FRS) [22]. The indicators were entered the Formula, such as age, total cholesterol (TC), high-density lipoprotein cholesterol (HDL-C), upper arm systolic pressure, current smoker (yes, no) and diabetes (yes, no), which provided sex-specific results. For simplicity, vascular age values of “ > 80” and “ < 30” were replaced with 80 years and 30 years old, respectively [23].

### Covariates

Trained interviewers collected data on sociodemographic characteristics, lifestyle behaviors, work-related details, and medical history using structured questionnaires (Appendix 1). Seniority was defined as the number of years an individual had been employed at this ferro-manganese refinery. Current smoker was defined as those who smoked more than one time per day for a duration exceeding half of a year. Current drinker was defined as those who drank at least one time in a week for exceeding half of a year. Hypertension was identified if any of these conditions were met: (1) a doctor had diagnosed the individual with hypertension; (2) the person was currently taking medication for high blood pressure; (3) systolic blood pressure was 140 mmHg or higher; or (4) diastolic blood pressure was 90 mmHg or higher. Diabetes was defined as a self-reported diagnosis or current use of glucose-lowering medications.

### Statistical analysis

For categorical variables, demographic characteristics are displayed as frequencies and percentages, while continuous variables are represented by medians (25, 75th percentile). To evaluate the normality of continuous variables, we used the Kolmogorov–Smirnov test. We conducted log10-transformation of the metal concentrations and baPWV values to improve normality. Additionally, to assess the correlations among metals, a correlation heatmap was constructed on the basis of Spearman rank correlation coefficients.

In the single-metal model, a generalized linear model (GLM) with gaussian distribution and identity link function was utilized to assess the association between specific metal and baPWV. The covariates were entered into GLM model simultaneously unless otherwise stated, such as age (continuous), gender (male, female), seniority (continuous), BMI (continuous), current smoker (yes, no), current drinker (yes, no), hypertension (yes, no), diabetes (yes, no), TC (continuous), TG (continuous), HDL-C (continuous) and LDL-C (continuous).

To construct a multi-metal model, least absolute shrinkage and selection operator (LASSO) regression was utilized. After performing tenfold cross-validation, we selected metals associated with baPWV based on the penalty parameter (λ), which resulted in the minimum mean squared error (MSE) [24]. Weighted quantile sum (WQS) regression was used to estimate the combined effect of the metals identified via LASSO regression. This model operates under the assumption that the relationships between all exposures and the outcome are in the same direction [25]. The model constructed a weighted index that assessed the combined effect of metal mixtures on baPWV, thereby identifying key metals. Further, the restricted cubic spline (RCS) was utilized to estimate potential nonlinear associations setting three knots at 10th, 50th and 90th. To further construct a multi-metal model, a GLM was used to evaluate the associations between metals selected by LASSO regression and baPWV.

In addition, GLM was performed to evaluate the associations between blood metals and vascular age. To avoid duplicating the variables included in the calculation of vascular age, the models were adjusted for seniority (continuous), BMI (continuous), current drinker (yes, no), hypertension status (yes, no), TG (continuous) and LDL-C (continuous). Furthermore, the mediating role of vascular age on the relationship between metal and baPWV were assessed via mediation analysis. We also employed GLM to assess the joint and interactive effect of statistically specific metal with age, gender, BMI, seniority, smoking status and drinking status.

All analyses and visualizations were performed in R software (version 4.3.0). For implementing LASSO regression, WQS regression, RCS analysis, and mediation analysis, the R packages 'glmnet', 'gWQS', 'rms' and 'mediation' were utilized, respectively. A two-tailed *P*-value under 0.05 was considered statistically significant.

## Results

### Characteristics of the study population

Table 1 outlines demographic information of the participants (*n* = 431). The median age of the participants was 46.00 years, with 70.30% being male. Most were married and possessed a high school education or above. The median for seniority and BMI were 24.83 years and 24.20 kg/m^2^, respectively. Among the participants, 188 (43.62%) were current smokers and 100 (23.20%) were current drinkers. The median of TC, TG, HDL-C and LDL-C were 5.74 mmoL/L, 1.49 mmoL/L, 1.46 mmoL/L, 3.64 mmoL/L, respectively. And the median of vascular age was 54.00 years.

**Table 1:** The characteristics of the participants from the manganese-exposed workers healthy cohort (*n* = 431)

Variables	*n* (%) or median (*P*25, *P*75)
Age, years	46.00 (43.00, 49.00)
Gender	
Male	303 (70.30)
Female	128 (29.70)
Seniority, years	24.83 (17.17, 26.42)
BMI, kg/m^2^	24.20 (21.90, 26.60)
Ethnicity	
Han	171 (39.68)
Zhuang	244 (56.61)
Other	16 (3.71)
Education	
Middle school or lower	165 (38.28)
High school or higher	266 (61.72)
Marital status	
Single/widow	80 (18.56)
Married/cohabited	351 (81.44)
Current smoker	
Yes	188 (43.62)
No	243 (56.38)
Current drinker	
Yes	100 (23.20)
No	331 (76.80)
Hypertension	
Yes	160 (37.12)
No	271 (62.88)
Diabetes	
Yes	4 (0.93)
No	427 (99.07)
TC, mmol/L	5.74 (4.97, 6.51)
TG, mmol/L	1.49 (1.04, 2.33)
HDL-C, mmol/L	1.46 (1.24, 1.75)
LDL-C, mmol/L	3.64 (3.03, 4.34)
baPWV, m/s	15.47 (14.09, 17.03)
Vascular age, years	54.00 (45.00, 64.00)

*Abbreviations*: *BMI* body mass index, *TC* total cholesterol, *TG* triglyceride, *HDL-C* high density lipoprotein cholesterol, *LDL-C* low density lipoprotein cholesterol, *baPWV* brachial-ankle pulse wave velocity

Figure S1 shows the distribution of baPWV by different demographic characteristics. Older age (≥ 46 years), male, higher BMI (≥ 24 kg/m^2^), longer seniority (≥ 24.83 years), current smoker, and current drinker exhibited significantly higher baPWV values.

Table S1 provides the concentrations of blood metals. Among 12 metals, the concentration of Fe was the highest, and the concentration of Co was the lowest. Spearman coefficients for log10- transformation metals revealed the strongest correlation between Ti and Ca (r_s_ = 0.50). Other metals demonstrated weak to moderate correlations (r_s_ = −0.40 ~ 0.45) (Figure S2).

### Association of single metal and baPWV

**Fig. 1:**
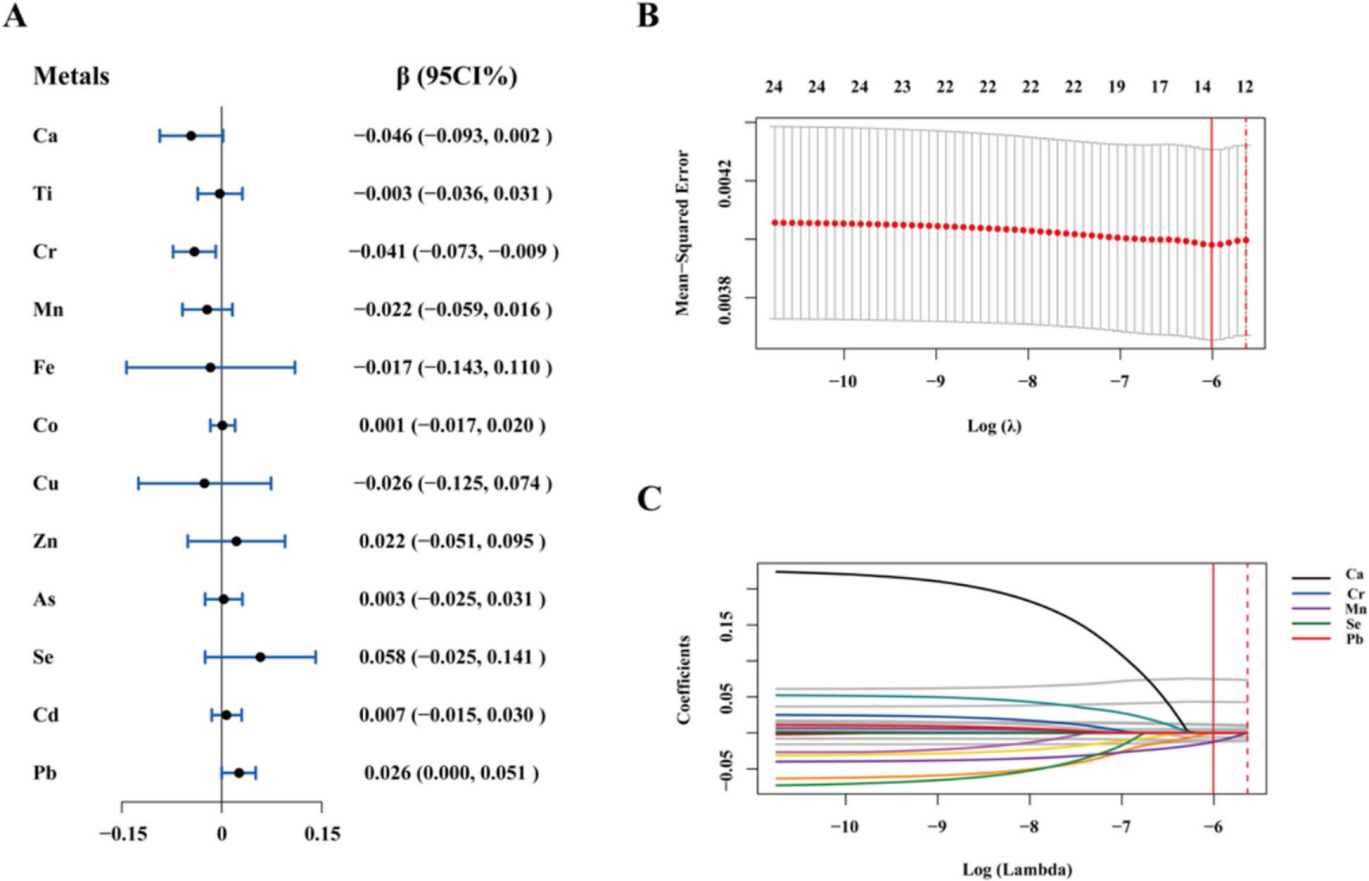
**A** Associations between individual blood metals with baPWV. (B—C) Screening of high-risk blood metals associated with baPWV by LASSO regression. Note: (**B**) shows the variation of mean square error (MSE) with log λ, and (**C**) shows the model screening path based on the log λ. With the increase of log λ, the more regression coefficients of metals were penalized to zero. The red solid line represents the log λ of minimum MSE, and the red dotted line represents the log λ of minimum MSE of a standard deviation. All models adjusted for age, sex, seniority, BMI, smoking status, drinking status, hypertension, diabetes, TC, TG, HDL-C, and LDL-C

Figure 1A demonstrates the relationship between individual blood metal and baPWV. In the continuous model, a negative association was observed between Cr and baPWV (β = −0.041, *P* = 0.014). Conversely, Pb exhibited a significantly positive association with baPWV (β = 0.026, *P* = 0.048). Furthermore, in the categorical model, baPWV decreased as the Cr increased, and compared with those in the first tertile, β values were −0.006 and −0.016 for the second and third tertiles, respectively (*P*_trend_ = 0.030). The baPWV increased as the Pb increased, and compared with those in the first tertile, β values were 0.012 and 0.016 for the second and third tertiles, respectively (*P*_trend_ = 0.085) (Table S3).

### Associations between multi-metal and baPWV

**Fig. 2:**
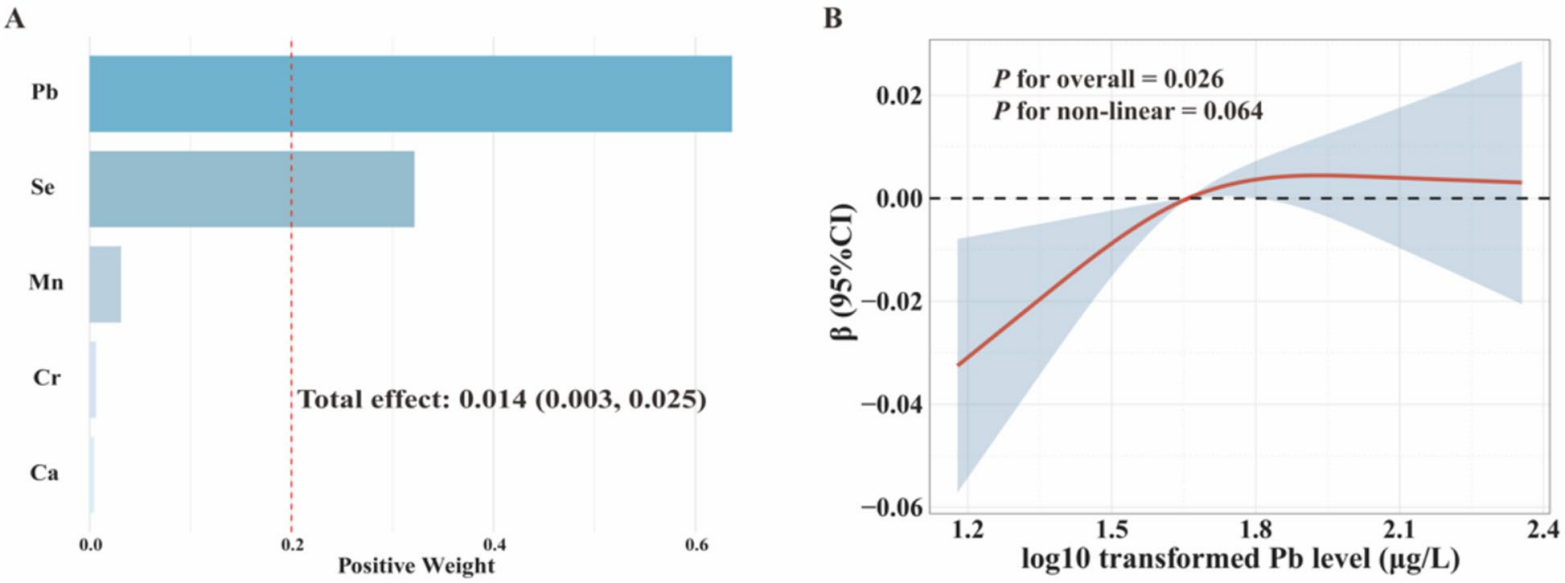
**A** Weights of individual blood metals in the positive WQS model for baPWV. **B** Associations of Pb concentration with baPWV based on restricted cubic splines. Solid and dashed lines represent the predicted values and 95% confidence intervals. Knots were placed at the 10th, 50th, and 90th percentiles of the log10-transformed Pb distribution, with the 50th percentile set as the reference. All models adjusted for age, gender, seniority, BMI, smoking status, drinking status, hypertension, diabetes, TC, TG, HDL-C, and LDL-C

Figure 1B&C shows that Ca, Cr, Mn, Se and Pb were identified as significant predictors of baPWV in LASSO regression, where the MSE was 0.004. The associations between the metal mixture (Ca, Cr, Mn, Se, Pb) and baPWV are shown in Fig. 2A and Figure S3. The baPWV increased by 0.014 m/s for each unit increment in the WQS index of the metal mixture, with Pb providing the greatest contribution (weight = 0.64). As depicted in Fig. 2B, a linear and positive dose–response relationship was revealed between Pb and baPWV (*P* for overall = 0.026, *P* for nonlinear = 0.064). Moreover, the GLM revealed a significantly negative correlation between Cr and baPWV [β (95% CI): −0.035 (−0.068, −0.002)] and a significantly positive correlation between Pb and baPWV [β (95% CI): 0.031 (0.005, 0.057)] (Figure S4).

### Associations between metals and vascular age

In the continuous variable model, each unit increase in log10-transformation As, Se, Cd, and Pb was associated with an increase in vascular age, the corresponding β values were 0.036, 0.154, 0.092 and 0.082, respectively (all *P* < 0.05). Each unit increase in log10-transformed Ca or Mn was associated with a decrease in vascular age, and the corresponding β values were −0.083 and −0.052, respectively (both *P* < 0.05). The significant associations mentioned above were further confirmed in the categorical variable model (all *P*_trend_ < 0.05) (Table 2).

**Table 2:** Association between individual blood metals with vascular age

Metals	β (95% CI)	Tertiles			
		**β**_**T1**_** (95% CI)**	**β**_**T2**_** (95% CI)**	**β**_**T3**_** (95% CI)**	***P***_**trend**_
Ca	−0.083 (−0.136, −0.030)	Reference	−0.012 (−0.029, 0.005)	−0.024 (−0.041, −0.007)	0.005
Ti	−0.036 (−0.073, 0.001)	Reference	−0.013 (−0.029, 0.004)	−0.014 (−0.031, 0.003)	0.095
Cr	−0.032 (−0.069, 0.005)	Reference	0.009 (−0.007, 0.026)	−0.007 (−0.024, 0.009)	0.379
Mn	−0.052 (−0.093, −0.011)	Reference	−0.019 (−0.036, −0.002)	−0.027 (−0.044, −0.011)	0.001
Fe	0.062 (−0.082, 0.206)	Reference	−0.011 (−0.028, 0.006)	0.002 (−0.014, 0.019)	0.953
Co	−0.014 (−0.034, 0.006)	Reference	0.006 (−0.011, 0.023)	−0.011 (−0.027, 0.006)	0.146
Cu	0.042 (−0.073, 0.156)	Reference	−0.003 (−0.020, 0.013)	0.008 (−0.009, 0.024)	0.279
Zn	0.042 (−0.041, 0.124)	Reference	−0.010 (−0.027, 0.007)	0.004 (−0.013, 0.021)	0.585
As	0.036 (0.004, 0.067)	Reference	0.017 (0.000, 0.034)	0.020 (0.003, 0.037)	0.018
Se	0.154 (0.061, 0.247)	Reference	0.007 (−0.010, 0.024)	0.022 (0.005, 0.039)	0.012
Cd	0.092 (0.077, 0.107)	Reference	0.034 (0.019, 0.049)	0.088 (0.072, 0.103)	< 0.001
Pb	0.082 (0.057, 0.107)	Reference	0.040 (0.023, 0.057)	0.058 (0.041, 0.075)	< 0.001

The models adjusted for seniority, BMI, drinking status, hypertension, TG and LDL-C

### Association between vascular age and baPWV

The association between vascular age and baPWV is presented in Table 3. In the rude model, we observed a significantly positive correlation between vascular age and baPWV [β (95% CI): 0.283 (0.225, 0.341)]. Furthermore, the positive association between vascular age and baPWV remained significant [β (95% CI): 0.225 (0.144, 0.307)].

**Table 3:** Association of vascular age with baPWV

Models	β (95% CI)	*P*.value
Model1	0.283 (0.225, 0.341)	< 0.001
Model2	0.225 (0.144, 0.307)	< 0.001

Model 1 was unadjusted

Model 2 adjusted for seniority, BMI, drinking status, hypertension, TG, LDL-C

### Mediation analysis

**Fig. 3:**
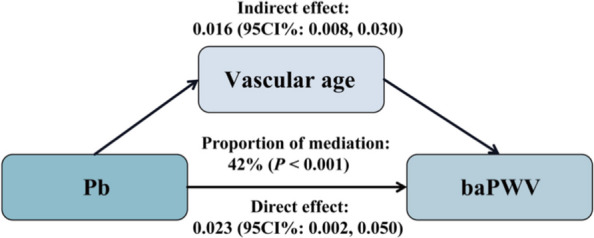
The mediating role of vascular age in associations of Pb exposure with baPWV. Association between Pb and baPWV was adjusted for age, gender, seniority, BMI, smoking status, drinking status, hypertension, diabetes, TC, TG, HDL-C, and LDL-C. Associations between metals and vascular age, as well as between vascular age and baPWV, were adjusted for seniority, BMI, drinking status, hypertension, TG, and LDL-C

An analysis of the relationships among metals, baPWV and vascular age, revealed that out of the 12 metals investigated, only Pb was significantly associated with both baPWV and vascular age. We further explored the mediating role of vascular age in the relationship between Pb exposure and baPWV. It demonstrated that vascular age served as a partial mediator in the relationship between Pb and baPWV, with a mediation proportion of 42%. The β values (95% CI) for the direct and indirect effects were 0.023 (0.002, 0.050) and 0.016 (0.008, 0.030), respectively (Fig. 3).

### Joint and interactive effects

**Fig. 4:**
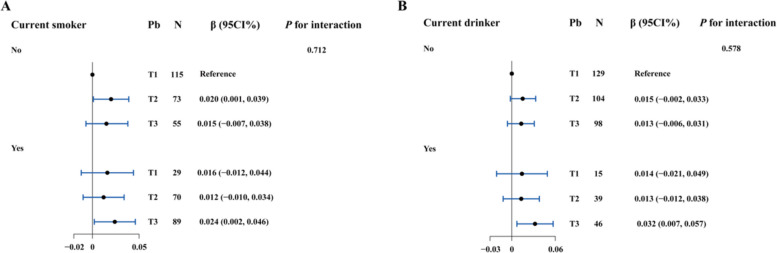
The joint and interactive effects of Pb exposure with smoking status and drinking status on changes in baPWV. The models adjusted for age, gender, seniority, BMI, smoking status, drinking status, hypertension, diabetes, TC, TG, HDL-C, and LDL-C

Figure 4 shows that compared with individuals who did not smoke and had blood Pb concentrations in the first tertile, those who smoked and had Pb concentrations in the third tertile presented higher baPWV levels [β (95% CI): 0.024 (0.002, 0.046)]. Moreover, compared to non-drinkers with low Pb exposure, drinkers with high Pb exposure presented a significant increase in baPWV [β (95% CI): 0.032 (0.007, 0.057)]. However, smoking or drinking status did not show any significant modified effect on the association between Pb and baPWV (both *P*_interaction_ > 0.05).

Figure S5 demonstrates the joint and interactive effects of Pb exposure with other variables on baPWV changes. Individuals aged ≥ 46 years (vs. < 46 years) or with BMI ≥ 24 kg/m^2^ (vs. < 24 kg/m^2^) in the highest blood Pb tertile showed significantly higher baPWV levels (β = 0.031 and 0.024, respectively; both *P* < 0.05). Among individuals with seniority < 24.83 years, higher baPWV levels were observed in those whose blood Pb concentration in the third tertile, compared those in the first tertile [β (95% CI): 0.028 (0.005, 0.051)]. Only gender had a marginally significant modified effect on the association between Pb exposure and baPWV (*P*_interaction_ = 0.055).

## Discussion

This study explored the associations between 12 blood metals and atherosclerosis and the potential mechanism based on MEWHC. We found that elevated blood Pb was significantly associated with increased baPWV and was identified as the primary contributor in the mixture analysis. Vascular age, which was positively correlated with Pb, acted as a key mediator in this relationship. Furthermore, the association between Pb and baPWV could be modified by factors such as age, BMI, smoking status, and drinking status. These findings provide crucial insights for formulating future strategies to prevent atherosclerosis.

Our findings suggested that increased blood Pb level was linked to atherosclerosis, which is in line with earlier reports [17, 26]. Evidence from cross-sectional studies indicated that Pb exposure was positively associated with risk of atherosclerosis among fast food workers in Iraq [27] and in a Swedish population-based cohort, even in those with low level [28–30]. Experimental evidence indicates that Pb exposure promotes atherosclerosis through multiple interacting pathways. Key mechanisms include: (1) inducing oxidative stress-by increasing reactive oxygen species (ROS) generation and impairing antioxidant defenses (e.g., glutathione synthesis and superoxide dismutase activity), leading to lipid oxidation and plaque formation [17, 31]; (2) triggering cardiovascular inflammatory responses and endothelial injury (e.g., elevated levels of soluble adhesion molecules), which further contribute to endothelial dysfunction and vascular structural remodeling [32–34]; and (3) disrupting calcium homeostasis in cardiomyocytes, thereby affecting cardiac contractility and increasing susceptibility to cardiovascular disorders [32, 35]. These processes interact synergistically to drive the progression of atherosclerosis.

Here, we observed a negative association between Cr and atherosclerosis, which aligns with the results from previous research [36, 37]. A study of elderly individuals in Chinese communities revealed a negative association between Cr and 10-year atherosclerotic cardiovascular disease risk [38]. Similarly, a review showed a steady inverse relationship between Cr level in serum, urine, whole blood as well as toenail and risk of atherosclerotic disease [39]. However, two studies also have shown that Cr was related to the formation of atherosclerotic plaques [40, 41]. This difference may stem from the different forms of Cr. Cr is usually found in two forms, namely trivalent [Cr (III)] and hexavalent [Cr (VI)]. Cr (III) is the most common form found in the natural environment and plays a beneficial role in the metabolism of glucose, lipids, and proteins. In contrast, Cr (VI) is produced primarily through industrial processes and is recognized for its high toxicity [17]. Studies have shown that Cr (III) may exert protective effects through multiple mechanisms. Specifically, Cr (III) can improve the lipid profile by increasing the expression of several genes, including PPARs-ϒ, GLUT 1, 3 and 4, and LDLR, and reduce the inflammatory level by inhibiting NF-kB activation. In addition, it may alleviate oxidative stress by reducing and eliminating ROS and activating detoxification related enzymes [36]. Although some mechanisms have been proposed, there are still inconsistencies on the relationship between Cr and atherosclerosis, and the specific underlying mechanism requires further rigorous research and verification.

Our research showed that vascular age was positively associated with baPWV, which was consistent with prior studies [42, 43]. Vascular age serves as an important indicator for evaluating vascular aging. Consistent findings from population-based research highlight vascular aging as a significant risk factor for the development of arteriosclerosis [44, 45]. Research has revealed that in the plaque tissues of patients with atherosclerosis, senescent endothelial cells, progenitors and vascular smooth muscle cells all exhibited senescent characteristics such as extensive expression of SA-β-galactosidase, telomeres shortening and activation of p16/p21 [46]. The subendothelial space in atherosclerotic mouse models also presents highly expressed aging phenotypic factors, including SA-β-galactosidase activity, p16INK4A expression, and senescence-associated secretory phenotype (SASP) factor [47]. In addition, a review suggested that vascular age contributes to the development of atherosclerosis through several mechanisms, including endothelial barrier dysfunction/damage, diastolic dysfunction and sclerosis, clotting equilibrium disruption, and inflammatory cell infiltration [14]. These mechanisms collectively highlight the pivotal role of vascular aging in the onset and advancement of atherosclerosis.

We observed that vascular age mediated 42% of the associations between Pb and baPWV. Vascular age may mediate the relationship between Pb and arteriosclerosis through various mechanisms. Research has shown that elevated Pb levels can boost ROS production and reduce antioxidant molecule levels, thereby inducing oxidative stress and impairing vascular structure and function, which accelerated vascular aging [48]. The resulting cellular damage (such as lipid peroxidation, DNA lesions and epigenetic alterations) compromises cellular integrity, alters the intravascular environment and ultimately promotes the occurrence and progression of atherosclerosis [32, 49]. Atherosclerosis development involves vascular inflammation, a process regulated by the NF-κB pathway [50]. Research has demonstrated that occupational exposure to Pb was associated with increased levels of inflammatory cytokines such as TNF-α, IL-4, and IL-10, indicating that Pb can trigger systemic inflammatory responses [51]. Persistent chronic inflammation, while activating the immune system, also damaged the vascular endothelium, and disrupted the cytokine network and homeostasis, thereby promoting the occurrence of “Inflamma-aging” [52]. This inflammation-driven vascular aging is characterized by impaired endothelial barrier integrity, reduced vasodilatory capacity, increased inflammatory cell infiltration, and enhanced plaque formation, ultimately promoting the onset and advancement of atherosclerosis [53].

Joint effects analysis revealed that current smokers, current drinkers, older individuals, and those with higher BMI values with elevated Pb exposure presented higher baPWV levels. These findings suggesting a synergistic interaction that promotes atherosclerosis, consistent with existing evidence. Smoking, drinking, older age, and higher BMI are well-established risk factors for CVD and are strongly linked to atherosclerosis [54]. Moreover, studies have shown that individuals with a history of smoking or drinking have relatively high Pb levels, suggesting that these habits may enhance the toxic effects of Pb [29]. Moreover, tobacco smoke contains Pb, which can increase the Pb burden in the body [55]. Alcohol also aggravated physiological stress, resulting in increases in blood pressure, heart rate and inflammatory markers [56]. With increasing age, the body’s antioxidant capacity gradually decreases, susceptibility to environmental hazards increases, and individuals become more prone to inflammatory-aging reactions, accelerating damage to vascular structures and functional degeneration [52]. In addition, a higher BMI is an important proinflammatory factor that can exacerbate systemic inflammatory responses and induce endothelial dysfunction, thereby promoting the occurrence and development of atherosclerosis [57]. Therefore, to mitigate the impact of Pb on atherosclerosis, it is necessary to actively control these risk factors and improve unhealthy lifestyles, which provide potential strategies.

Previous studies have confirmed the existence of gender difference in Pb accumulation. The higher hematocrit in males makes it easier for Pb to bind with red blood cells and accumulate in the body. However, Pb exposure itself can also have gender-specific effects on red blood cell parameters [58]. This inverse regulation may attenuate the accumulation differences, leading to a marginally significant moderating effect of gender in the association between Pb exposure and baPWV. Epidemiological evidence indicates that males typically have higher Pb burdens [59], and differences in endocrine, genetic, biochemical, and environmental factors collectively contribute to varying vulnerabilities to toxins between genders. This may make male exposure more likely to result in more significant vascular aging [60]. From a physiological mechanism perspective, gender heterogeneity in this association is mediated by hormonal regulation. Specifically, Pb exposure interferes with estrogen signaling and accelerates its depletion, thereby weakening vascular protection, while androgens may upregulate lead uptake in males and exacerbate oxidative stress, leading to increased arterial stiffness [58, 61].

This study has several strengths. First, we systematically assessed the relationships of exposure to metal mixtures with atherosclerosis, reflecting the exposure situation in the real world. Second, the mediating role of vascular age in the relationship between Pb exposure and arteriosclerosis was revealed, which helps reveal the potential biological mechanism pathway involved. Third, the influence of general demographic characteristics on the relationship between Pb and arteriosclerosis was further explored, suggesting that targeting these factors may serve as a potential strategy for risk reduction, with important public health implications. However, this study has several limitations. First, cross-sectional studies may restrict causal inference and produce reverse causality, longitudinal or experimental studies are needed to confirm our findings. Second, metal exposure was assessed using single blood sample testing, which may not accurately reflect long-term exposure. Third, whole blood is not necessarily suitable as an exposure marker for all 12 metals. And multiple imputation was not used to handle missing data which may cause bias. Additionally, the study population consisted of occupational workers, limiting the generalizability of the findings to the general population. Last, vascular aging and arteriosclerosis can be evaluated through more indicators to reflect their characteristics more comprehensively.

## Conclusions

Our study revealed that exposure to lead (Pb) was significantly and positively associated with baPWV, showing higher Pb level related to higher atherosclerosis risk. Furthermore, vascular age acted as a positive mediator of this relationship. Moreover, healthy habits and BMI may help mitigate the harmful effects of Pb. Further prospective and mechanistic studies are recommended to corroborate the findings and clarify the elusive mechanism.

## Supplementary Information

Supplementary Material 1: Table S1 Blood metal concentrations in 431 participants. Table S2 Quality control results for the detection of metals by ICP-MS. Table S3 Association between individual blood metals and baPWV. Figure S1 Distribution of baPWV by different demographic characteristics. (A) Distribution of baPWV by age groups. (B) Distribution of baPWV by gender. (C) Distribution of baPWV by BMI groups. (D) Distribution of baPWV by seniority groups. (E) Distribution of baPWV by smoking status. (F) Distribution of baPWV by drinking status.Note: The Mann-Whitney U test was used for comparisons between two independent groups. Figure S2 Correlation map of blood metals (log10) among participants. Figure S3 Weights of individual blood metals in the negative WQS model for baPWV. The model adjusted for age, gender, seniority, BMI, smoking status, drinking status, hypertension, diabetes, TC, TG, HDL-C, and LDL-C. Figure S4 Associations of multiple blood metals with changes in baPWV. Note: For panel A, blood metals were included in the generalized linear regression models as continuous variables. For panel B, blood metals were analyzed as categorical variables (tertiles). *P* for trend across tertiles was calculated by modeling the median value of each tertile (log10-transformed) as a continuous variable in the model. Generalized linear regression models were adjusted for age, gender, seniority, BMI, smoking status, drinking status, hypertension, diabetes, TC, TG, HDL-C, and LDL-C. Figure S5 The joint and interactive effects of Pb exposure with general demographic characteristics on changes in baPWV. (A) age groups; (B) gender; (C) BMI groups; (D) seniority groups.

Supplementary Material 2.

## Data Availability

Data will be accessible if requested reasonably.
