# Supplementary material for: The mediating role of vascular age in the association between blood metals and atherosclerosis from Manganese-exposed workers healthy cohort
Source: BMC Public Health. 2026 Jan 16;26:562. doi: 10.1186/s12889-026-26235-5 (PMC12895618; doi:10.1186/s12889-026-26235-5)
Supplement: Supplementary file 1 — Supplementary Material 1: Table S1 Blood metal concentrations in 431 participants. Table S2 Quality control results for the detection of metals by ICP-MS. Table S3 Association between individual blood metals and baPWV. Figure S1 Distribution of baPWV by different demographic characteristics. (A) Distribution of baPWV by age groups. (B) Distribution of baPWV by gender. (C) Distribution of baPWV by BMI groups. (D) Distribution of baPWV by seniority groups. (E) Distribution of baPWV by smoking status. (F) Distribution of baPWV by drinking status.Note: The Mann-Whitney U test was used for comparisons between two independent groups. Figure S2 Correlation map of blood metals (log10) among participants. Figure S3 Weights of individual blood metals in the negative WQS model for baPWV. The model adjusted for age, gender, seniority, BMI, smoking status, drinking status, hypertension, diabetes, TC, TG, HDL-C, and LDL-C. Figure S4 Associations of multiple blood metals with changes in baPWV. Note: For panel A, blood metals were included in the generalized linear regression models as continuous variables. For panel B, blood metals were analyzed as categorical variables (tertiles). P for trend across tertiles was calculated by modeling the median value of each tertile (log10-transformed) as a continuous variable in the model. Generalized linear regression models were adjusted for age, gender, seniority, BMI, smoking status, drinking status, hypertension, diabetes, TC, TG, HDL-C, and LDL-C. Figure S5 The joint and interactive effects of Pb exposure with general demographic characteristics on changes in baPWV. (A) age groups; (B) gender; (C) BMI groups; (D) seniority groups. [file 12889_2026_26235_MOESM1_ESM.docx]

| Table S1 Blood cell metal concentrations in 431 participants. |
| --- |
| \| Variables \| LOD \| >LOD% \| *P*05 \| *P*25 \| *P*50 \| *P*75 \| *P*95 \| \| --- \| --- \| --- \| --- \| --- \| --- \| --- \| --- \| \| Ca, mg/L \| 1.933 \| 100.00 \| 7.87 \| 10.52 \| 12.59 \| 15.07 \| 20.84 \| \| Ti, μg/L \| 0.029 \| 100.00 \| 2.21 \| 3.39 \| 4.42 \| 5.52 \| 8.62 \| \| Cr, μg/L \| 0.005 \| 99.54 \| 0.23 \| 0.34 \| 0.41 \| 0.51 \| 0.84 \| \| Mn, μg/L \| 0.003 \| 100.00 \| 13.53 \| 19.41 \| 24.59 \| 31.14 \| 47.66 \| \| Fe, mg/L \| 6.676 \| 100.00 \| 1154.86 \| 1288.84 \| 1421.38 \| 1506.5 \| 1612.69 \| \| Co, μg/L \| <0.001 \| 100.00 \| 0.06 \| 0.10 \| 0.14 \| 0.25 \| 0.61 \| \| Cu, μg/L \| 0.007 \| 100.00 \| 630.19 \| 688.64 \| 729.97 \| 808.08 \| 975.14 \| \| Zn, mg/L \| 0.059 \| 100.00 \| 7.53 \| 8.84 \| 9.86 \| 11.08 \| 14.04 \| \| As, μg/L \| 0.002 \| 100.00 \| 1.03 \| 1.51 \| 1.90 \| 2.43 \| 4.19 \| \| Se, μg/L \| 0.050 \| 100.00 \| 154.57 \| 177.75 \| 195.5 \| 223.06 \| 272.58 \| \| Cd, μg/L \| <0.001 \| 100.00 \| 0.92 \| 1.72 \| 3.33 \| 9.38 \| 18.46 \| \| Pb, μg/L \| 0.001 \| 100.00 \| 18.56 \| 30.67 \| 45.63 \| 67.58 \| 155.04 \| |
| Abbreviations: LOD, limit of detection. |

Table S2 Quality control results for the detection of metals by ICP-MS

| Metals  (μg/L) |  | Seronorm^TM^ Trace Elements  Whole Blood RUO Level-1  (n = 19) | |  | Seronorm^TM^ Trace Elements  Whole Blood RUO Level-2  (n = 19) | |  | Seronorm^TM^ Trace Elements  Whole Blood RUO Level-3  (n = 19) | |
| --- | --- | --- | --- | --- | --- | --- | --- | --- | --- |
|  |  | Certified value（μg/L） | Measured value（μg/L） |  | Certified value（μg/L） | Measured value（μg/L） |  | Certified value（μg/L） | Measured value（μg/L） |
| Ca |  | 11700~17600 | 15472.64 ± 1005.27 |  | 32000~48000 | 42913.61 ± 2102.13 |  | 49000~61000 | 62546.57 ± 3298.69 |
| Cr |  | 0.48~0.75 | 1.1 ± 0.55 |  | 7.9~11.8 | 10.2 ± 0.68 |  | 27.5~41.3 | 35.67 ± 2 |
| Mn |  | 13~19.6 | 16.01 ± 0.84 |  | 20.7~24.3 | 22.86 ± 1.03 |  | 26.2~39.4 | 32.91 ± 1.7 |
| Co |  | 0.16~0.24 | 0.36 ± 0.11 |  | 4~6.1 | 5.38 ± 0.33 |  | 7.9~11.9 | 10.44 ± 0.54 |
| Cu |  | 520~780 | 646.08 ± 41.8 |  | 830~1000 | 922.6 ± 57.78 |  | 1460~2200 | 1993.51 ± 107.94 |
| Zn |  | 3600~5400 | 4770.42 ± 251.27 |  | 5000~7500 | 5987.28 ± 285.66 |  | 5700~8600 | 6349.73 ± 308.59 |
| As |  | 2~3 | 2.29 ± 0.12 |  | 9.1~13.7 | 12.26 ± 0.76 |  | 19.4~29.2 | 26.53 ± 1.37 |
| Se |  | 47~87 | 85.21 ± 15.47 |  | 63~118 | 113.27 ± 16.13 |  | 115~214 | 220.74 ± 19.14 |
| Cd |  | 0.23~0.35 | 0.26 ± 0.03 |  | 4~6 | 4.98 ± 0.27 |  | 7.8~11.7 | 9.78 ± 0.34 |
| Pb |  | 8.2~12.4 | 9.08 ± 0.53 |  | 264~325 | 284.28 ± 13.91 |  | 345~519 | 443.48 ± 22.59 |

Table S3 Association between individual blood metals and baPWV.

| **Metals** | **Tertiles** | | | |
| --- | --- | --- | --- | --- |
|  | **β_T1_ (95% CI)** | **β_T2_ (95% CI)** | **β_T3_ (95% CI)** | ***P*_trend_** |
| Ca | Reference | -0.009 (-0.024, 0.005) | -0.007 (-0.022, 0.008) | 0.361 |
| Ti | Reference | 0.003 (-0.011, 0.018) | 0.002 (-0.013, 0.016) | 0.817 |
| Cr | Reference | -0.006 (-0.021, 0.008) | -0.016 (-0.031, -0.002) | 0.030 |
| Mn | Reference | -0.001 (-0.016, 0.014) | -0.004 (-0.020, 0.012) | 0.619 |
| Fe | Reference | 0.000 (-0.015, 0.015) | 0.000 (-0.015, 0.014) | 0.976 |
| Co | Reference | -0.003 (-0.018, 0.012) | 0.002 (-0.013, 0.017) | 0.725 |
| Cu | Reference | -0.001 (-0.016, 0.013) | -0.001 (-0.016, 0.013) | 0.874 |
| Zn | Reference | -0.002 (-0.016, 0.013) | -0.003 (-0.018, 0.011) | 0.652 |
| As | Reference | 0.006 (-0.009, 0.021) | 0.003 (-0.012, 0.019) | 0.663 |
| Se | Reference | 0.000 (-0.015, 0.014) | 0.007 (-0.007, 0.022) | 0.305 |
| Cd | Reference | -0.003 (-0.019, 0.012) | 0.007 (-0.015, 0.029) | 0.511 |
| Pb | Reference | 0.012 (-0.004, 0.028) | 0.016 (-0.002, 0.033) | 0.085 |


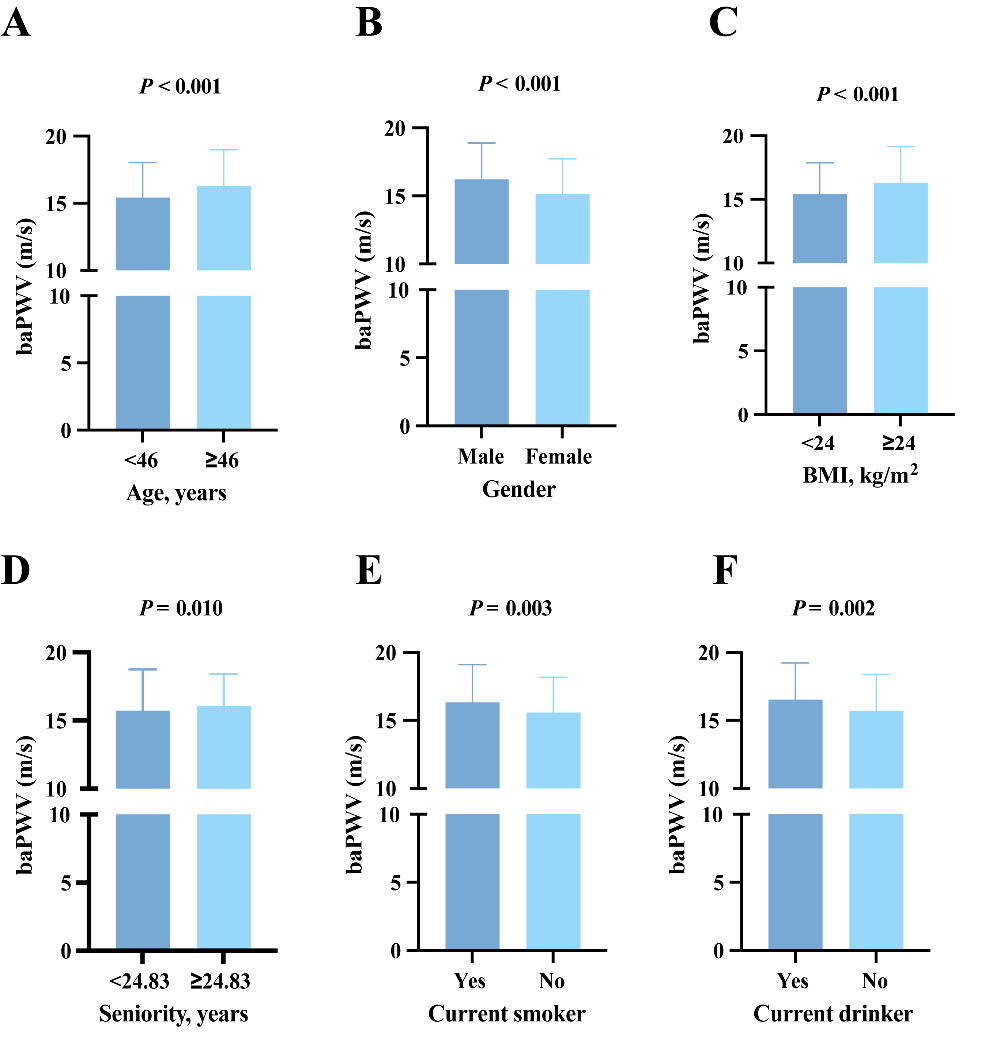


|  |
| --- |
| **Figure S1 Distribution of baPWV by different demographic characteristics.** (A) Distribution of baPWV by age groups. (B) Distribution of baPWV by gender. (C) Distribution of baPWV by BMI groups. (D) Distribution of baPWV by seniority groups. (E) Distribution of baPWV by smoking status. (F) Distribution of baPWV by drinking status.  **Note:** The Mann - Whitney U test was used for comparisons between two independent groups. |


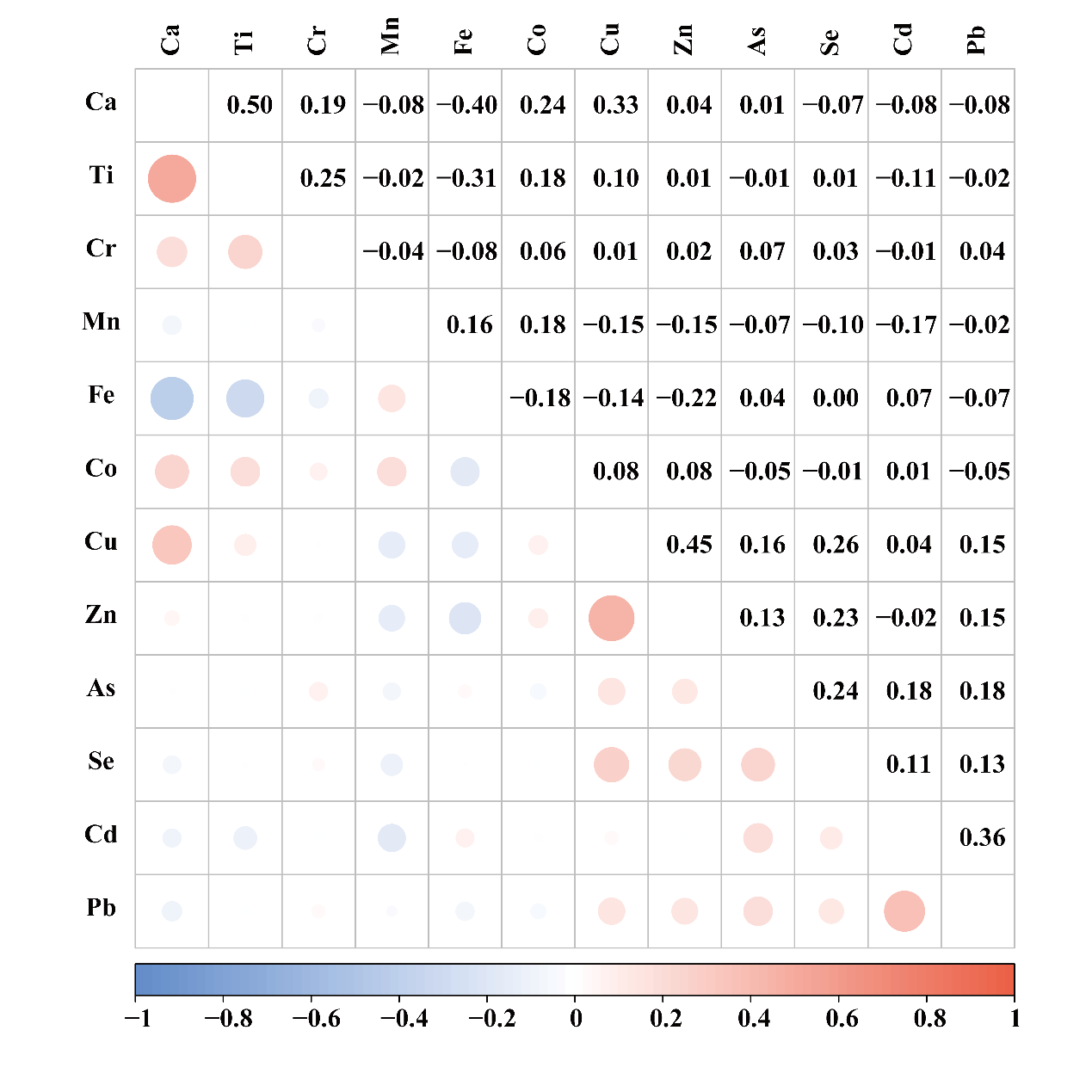


|  |
| --- |
| **Figure S2 Correlation map of blood metals (log10) among participants.** |


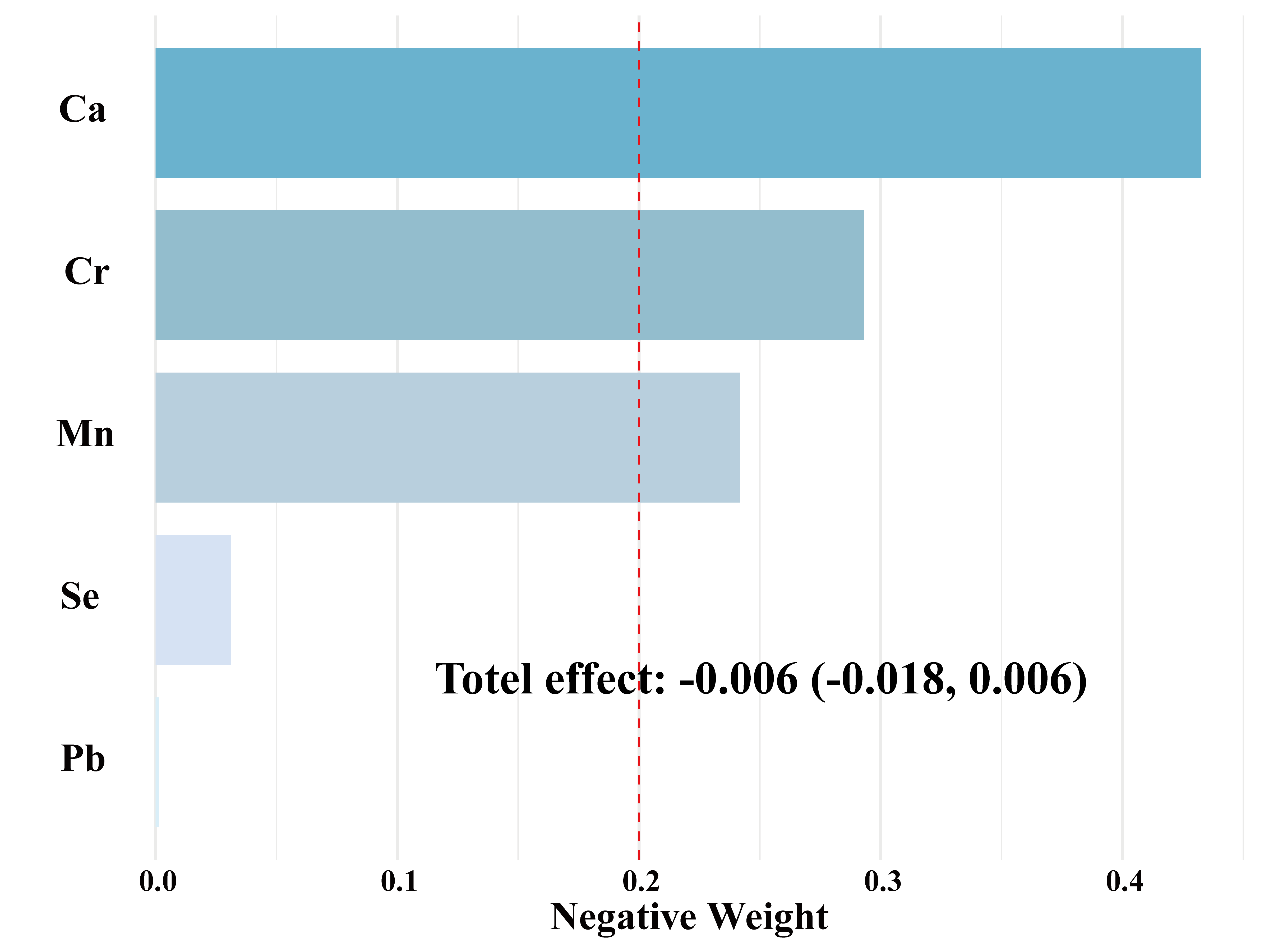


|  |
| --- |
| **Figure S3 Weights of individual blood metals in the negative WQS model for baPWV.**  The model adjusted for age, sex, seniority, BMI, smoking status, drinking status, hypertension, diabetes, TC, TG, HDL-C, and LDL-C. |


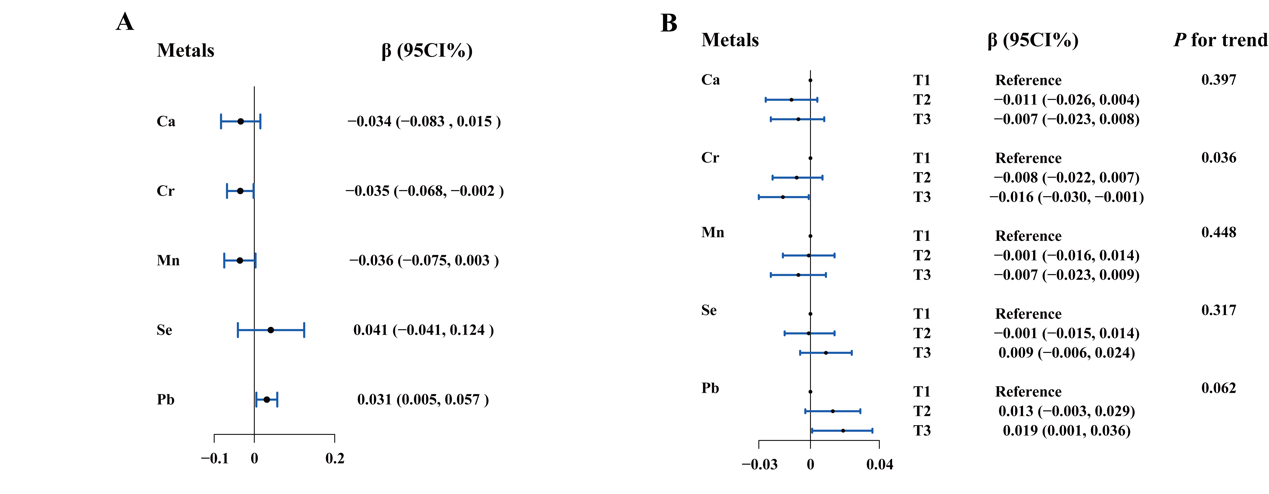


|  |
| --- |
| **Figure S4 Associations of multiple blood metals with changes in baPWV.**  **Note:** For panel A, blood cell metals were included in the generalized linear regression models models as continuous variables.  For panel B, blood cell metals were analyzed as categorical variables (tertiles). *P* for trend across tertiles was calculated by modeling the median value of each tertile (log10-transformed) as a continuous variable in the model. Generalized linear regression models were adjusted for age, sex, seniority, BMI, smoking status, drinking status, hypertension, diabetes, TC, TG, HDL-C, and LDL-C. |


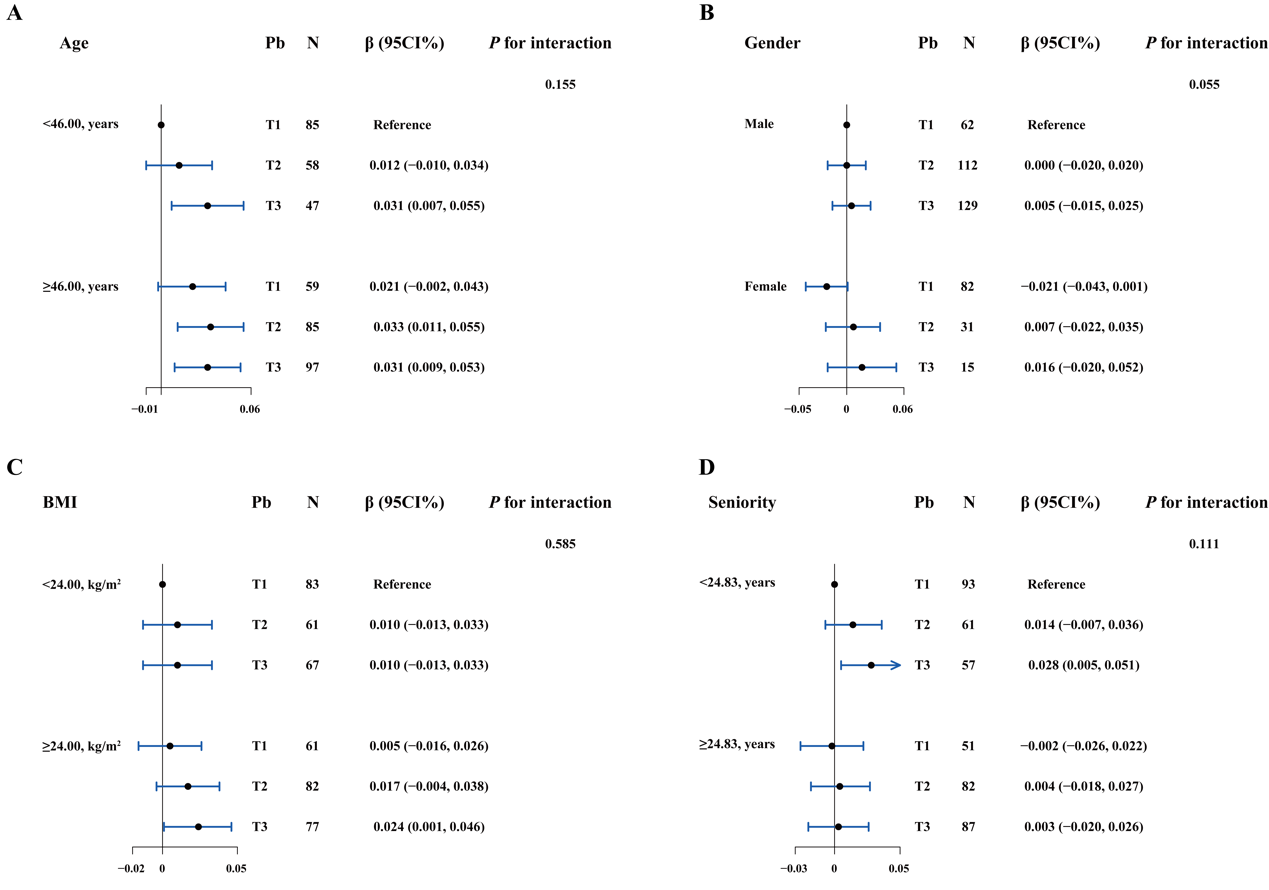


**Figure S5 The joint and interactive effects of Pb exposure with general demographic characteristics on changes in baPWV.** (A) age groups; (B) gender; (C) BMI groups; (D) seniority groups.
