# Supplementary material for: The mediating role of vascular age in the association between blood metals and atherosclerosis from Manganese-exposed workers healthy cohort
Source: BMC Public Health. 2026 Jan 16;26:562. doi: 10.1186/s12889-026-26235-5 (PMC12895618; doi:10.1186/s12889-026-26235-5)
Supplement: Supplementary file 2 — Supplementary Material 2. [file 12889_2026_26235_MOESM2_ESM.pdf]

Physical examination number: __________________

Current work location: _____________branch __________workshop

Stove number: _________ job category: ________

Note: ____

Physical examination Number Stickers

Baseline questionnaire form

I. Questionnaire Instructions

This questionnaire is designed to investigate the health status of occupational workers and the relevant factors affecting their health. The investigator should first read the instructions in detail, ask the respondents one by one according to the content of the questionnaire, and then fill out the questionnaire one by one according to the respondents' views or actual situation of each narrative. There are no right or wrong answers to the question, and the results of the questionnaire are analyzed as a whole without any individual presentation, and are absolutely confidential to the public, so please fill in the questionnaire truthfully according to the answers given by the occupational workers.

II. Filling Requirements

1. Please fill in the form with a black signature pen. **The handwriting should be neat and clear, not scribbled or blurred or randomly altered**.

2. Words or figures should be filled in the designated **line and "□"**.

3. Except for special instructions, no symbols should be filled in for options that are not logically necessary for the investigation.

4. Correction method after filling in the error: firstly, cross out the wrong words or figures with a double horizontal line, and fill in the correct words or figures above the crossed-out line, please do not scribble on the original figures or words.

Investigator (Signature): ________________________

Survey Completion Date: ____ Year ___ Month ___ Day

Guangxi Medical University

2021

Part I. Basic information

Serial number	Description	Fill in the answer sheet
A1	**Name and surname**	____________________
**A2 Identifying information and contact number**		
A2a	Identification number	_ _ _ _ _ _ _ _ _ _ _ _ _ _ _ _ _ _ (Write the numbers on the lines.)
A2b	Contact number	□□□□□□□□□□□
A3	**Gender**: 1. Male 2. Female	□
A4	**Height (cm)**	
A5	**Weight (kg)**	
A6	**Blood pressure (Systolic blood pressure/Diastolic blood pressure) (mmHg)**	**left /**<br>**right /**
A7	**Waist circumference (cm)**	
A8	**Hip circumference (cm)**	
A9	**Date of birth**	Solar calendar: □□□□Year□□Month□□Day
A10	**Permanent Address**	_______________county(city/district)______________township (street)<br>____ (address)
**A11 Place of origin** _________ Province ___________ County		
**A12 Ethnic group**: 1.Han 2.Zhuang 3.Other (Please write down the specific ethnicity) □ ____________		
A13	**Your personal education level is**:<br>1.Illiterate 2.Elementary School 3.Junior High School 4.Senior High school or Secondary Technical School 5.Junior College or Bachelor's Degree 6.Postgraduate or above	□
A14	**Your current marital status**:<br>1.Single 2.Married 3.Cohabitated 4.Widowed or divorced 5.Separated	□

Part II Personal Lifestyle Habits

Serial number	Description	Fill in the answer sheet
B1	**Have you ever smoked at least one cigarette a day for 6 months or more?** (One or more cigarettes per day for a continuous or cumulative period of 6 months or more is considered smoking; for silk smokers, 1 tael of silk = 60 paper cigarettes.)<br>1.Yes 2.No (skip to *B6*)	☐
B2	**At what age did you start smoking at least one cigarette a day?**	____________Years Old
B3	**When you were a regular smoker, how many cigarettes did you typically smoke on average per day?**	_____________Cigarettes/Day
B4	**Have you smoked regularly in the last 6 months?**<br>1.Yes (skip to *B6*) 2.No,quit smoking	☐
B5	**When did you start to quit smoking?**<br>1. three to six months ago 2. six months to one year ago<br>3. one to two years ago 4. two to five years ago<br>5. five years or more ago 6. can't remember	☐<br>Quitting time: From___Year ___Month<br>Note: ___________________
B6	**In the past year, has anyone smoked at home or at work while you were present?** 1.Yes 2. No	☐
C1	**Have you ever drunk alcohol?** (Drinking more than 5mL of alcohol on one occasion is considered to be drinking)<br>1.Yes 2.No (skip to *D1*)	☐
C2	**At what age did you start drinking regularly?** (Age of first drink over 5mL)	_____Years Old,<br>Drinking years:_____Years
C2a	How often have you been drinking alcohol for more than 6 consecutive months?<br>1.every day or almost every day 2.3-4 times a week<br>3.1-2 times a week	☐

	4.1-3 times per month 5.Less than 1 time per month	
**C3**	**What do you choose to drink on most occasions? (Single choice)**<br>1.Baijiu 2. Baijiu with medicinal liquor 3. Homemade rice wine 4. Homemade rice wine with medicinal liquor 5. Rice wine<br>6. Beer 7. Wine 8. Baijiu + Beer 9. Homemade rice wine + Beer 10. Other	□<br>other：_______
C3a	If you drink baijiu, approximately how much do you usually drink at a time?	__________tael/time（1 tael = 50mL）
C3b	If you drink baijiu with medicinal liquor, approximately how much do you usually drink at a time?	__________tael/time（1 tael = 50mL）
C3c	If you drink homemade rice wine, approximately how much do you usually drink at a time?	__________tael/time（1 tael = 50mL）
C3d	If you drink homemade rice wine with medicinal liquor, approximately how much do you usually drink at a time?	__________tael/time（1 tael = 50mL）
C3e	If you drink rice wine, approximately how much do you usually drink at a time?	__________tael/time（1 tael = 50mL）
C3f	If you drink beer, approximately how much do you usually drink at a time?	__________bottles/Time （1 bottle = 600mL ）
C3g	If you drink wine, approximately how much do you usually drink at a time?	__________tael/time（1 tael = 50mL）
C3h	If you drink baijiu + beer, approximately how much do you usually drink at a time?	Baijiu__tael/time,Beer___bottles/time
C3i	If you drink homemade rice wine + beer, approximately how much do you usually drink at a time?	Homemde rice wine__tael/time,<br>Beer___bottles/time
C3g	If you drink other alcohol, approximately how much do you usually drink at a time?	__________tael/time（1 tael = 50mL）
**C4**	**Have you been drinking alcohol regularly for the last 2 weeks?**	□

	1.Yes (skip to *C6*) 2.No, quit drinking	
C5	**When did you start to stop drinking?**<br>1. one month ago 2. one to six months ago<br>3. one year ago 4. two years ago 5. can't remember	☐<br>Quitting time: From___Year ___Month<br>Note: ____________________
C6	**Did you drink alcohol last night?** 1.Yes 2.No (skip to *D1*)	☐
C6a	What did you drink last night and how much did you drink? (See *C3* for options)	Type : ___; ___ tael or ___ bottles<br>___; ___ tael or ___ bottles
D1	**Did you drink tea in the last 6 months?** 1.Yes 2.No (skip to *E1*)	☐
D2	**Tea drinking frequency:**<br>1.daily or almost daily 2.3-4 times per week 3.1-2 times per week<br>4.1-3 times per month 5.Less than 1 time per month	☐
D3	**Which type of tea have you been drinking mostly in the last 6 months? (Single choice)**<br>1.green tea 2.black tea 3.clear tea (oolong tea)<br>4.about the same amount of black tea and green tea<br>5.about the same amount of clear tea and green tea<br>6.about the same amount of clear tea and black tea<br>7.other_________	☐ _________
E1	**Are you a vegetarian?**1.Yes 2.No	☐
E2	**How often have you consumed meat (all meat products) in the last 6 months:**<br>1.no meat at all 2. <1 time/week 3. 1-2 times/week 4. 3-4 times/week 5.almost 1 time/day<br>6. >1 time/day	☐
E3	**How often have you consumed eggs (e.g. eggs, duck eggs, etc.) in the last 6 months?**<br>1. <1 time/week 2. 1-2 times/week 3. 3-4 times/week 4.almost 1 time/day 5. >1 time/day	☐

E4a	How often have you consumed animal-based high-fat foods (e.g. fatty meat, fried, animal offal, etc.) in the last 6 months?<br>1. <1 time/week 2. 1-2 times/week 3. 3-4 times/week 4.almost 1 time/day 5.>1 time/day	☐
E4b	How often have you consumed plant-based high-fat foods (e.g. walnuts, sesame, peanuts, etc.) in the last 6 months?<br>1. <1 time/week 2. 1-2 times/week 3. 3-4 times/week 4.almost 1 time/day 5. >1 time/day	☐
**E5 Your dietary tastes?**		
E5a	Is it salty? 1.light 2.average 3.salty 4.very salty	☐
E5b	Is it spicy? 1.not spicy 2.average 3.on the spicy side 4.very spicy	☐
E5c	Is it on the sweet side? 1.not sweet 2.average 3.on the sweet side 4.very sweet	☐
**F1**	**Do you require shift work?** 1. Yes 2. No	☐
F1a	What kind of shift are you currently working?<br>1.day shift 2.medium shift 3.night shift 4.long day shift	☐
F1b	What shift were you working before you came for your physical examination?<br>1.day shift 2.medium shift 3.night shift 4.long day shift	☐
**F2**	**Your last commute to work before you come in for your physical examination**	Work start time:<br>___Month__Day__Hour___Minute<br>Work end time:<br>___Month__Day__Hour___Minute
**F3**	**Your work and rest schedule in the past year?**	Get up in the morning ：<br>______Hour ______Minute<br>Go to bed at night ：

		______Hour______Minute
F4	**Have you napped regularly in the last year?** 1.Yes 2.No	☐ When working :_____Hours When not working :_____ Hours
F5	**How was the quality of your sleep in the last year?** 1. good 2. average 3. poor 4. very poor, often with the help of sleeping pills	☐

Part III

3.1Medical history and family history

Disease Name	Do you suffer from this disease? (1. Yes 2. No)	First diagnosis time	Is the disease now under control? (1. Yes 2. No)	Relative with the disease (1. Yes 2. No)			
				children	siblings	father	mother
I1A1 Hypertension	☐	☐☐☐☐Years ☐☐Month☐☐Day	☐	☐	☐	☐	☐
I1A2 Coronary artery disease	☐	☐☐☐☐Years ☐☐Month☐☐Day	☐	☐	☐	☐	☐
I1A2-1 Type of coronary heart disease: 1. Angina pectoris 2. Acute myocardial infarction 3. Asymptomatic 4. Ischemic cardiomyopathic type 99. Unknown						☐☐	
I1A3 Stroke	☐	☐☐☐☐Years ☐☐Month☐☐Day	☐	☐	☐	☐	☐
I1A3-1 Type of stroke : 1. Cerebral hemorrhage 2. Cerebral infarction 99. Unknown						☐☐	
I1B1 Diabetes	☐	☐☐☐☐Years ☐☐Month☐☐Day	☐	☐	☐	☐	☐
I1B2 Thyroid Disease	☐	☐☐☐☐Years ☐☐Month☐☐Day	☐	☐	☐	☐	☐
I1B2-1 Types of thyroid disorders: 1. Thyroid 2. Hyperthyroidism 3. Hypothyroidism 99. Unknown						☐☐	

I1B3 Gout	☐	☐☐☐☐Years ☐☐Month☐☐Day	☐	☐	☐	☐	☐
I1C1 Fatty Liver Disease	☐	☐☐☐☐Years ☐☐Month☐☐Day	☐	☐	☐	☐	☐
I1C2 Hepatitis B	☐	☐☐☐☐Years ☐☐Month☐☐Day	☐	☐	☐	☐	☐
I1C3 Chronic Hepatitis	☐	☐☐☐☐Years ☐☐Month☐☐Day	☐	☐	☐	☐	☐
I1C4 Cirrhosis	☐	☐☐☐☐Years ☐☐Month☐☐Day	☐	☐	☐	☐	☐
I1D1 Prostatitis	☐	☐☐☐☐Years ☐☐Month☐☐Day	☐	☐	☐	☐	☐
I1E1 Rheumatoid Arthritis	☐	☐☐☐☐Years ☐☐Month☐☐Day	☐	☐	☐	☐	☐
I1F1 Iron Deficiency Anemia	☐	☐☐☐☐Years ☐☐Month☐☐Day	☐	☐	☐	☐	☐
I1F2 Thalassemia	☐	☐☐☐☐Years ☐☐Month☐☐Day	☐	☐	☐	☐	☐
I1G1 Schistosomiasis	☐	☐☐☐☐Years ☐☐Month☐☐Day	☐	☐	☐	☐	☐
I1H1 Chronic Gastritis	☐	☐☐☐☐Years ☐☐Month☐☐Day	☐	☐	☐	☐	☐
I1H2 Peptic Ulcer	☐	☐☐☐☐Years ☐☐Month☐☐Day	☐	☐	☐	☐	☐
I1H3 Other stomach problems	☐	☐☐☐☐Years ☐☐Month☐☐Day	☐	☐	☐	☐	☐
I1H3a Other stomach problems	____________________						
I1I1 Tumor	☐	☐☐☐☐Years ☐☐Month☐☐Day	☐	☐	☐	☐	☐

I1I1a Tumor Name	____________________						
Disease Name	Do you suffer from this disease? (1. Yes 2. No)	First diagnosis time	Is the disease now under control? (1. Yes 2. No)	Relative with the disease (1. Yes 2. No)			
				children	siblings	father	mother
I1J1 Other Diseases	____________	☐☐☐☐Years ☐☐Month☐☐Day	☐	☐	☐	☐	☐
I1J2 Other Diseases ________	____________	☐☐☐☐Years ☐☐Month☐☐Day	☐	☐	☐	☐	☐
I1J3 Other Diseases ________	____________	☐☐☐☐Years ☐☐Month☐☐Day	☐	☐	☐	☐	☐
I1J4 Other Diseases ________	____________	☐☐☐☐Years ☐☐Month☐☐Day	☐	☐	☐	☐	☐
I1J5 Other Diseases ________	____________	☐☐☐☐Years ☐☐Month☐☐Day	☐	☐	☐	☐	☐

I1K1Have you ever had surgery?		Surgery name	Surgical hospitals	Surgical time
1.Yes 2.No	☐			☐☐☐☐Years☐☐Month☐☐Day
1.Yes 2.No	☐			☐☐☐☐Years☐☐Month☐☐Day
1.Yes 2.No	☐			☐☐☐☐Years☐☐Month☐☐Day

3.2 Past medication history

Serial number	Description	Fill in the answer sheet
H1	Have you taken any medications in the last 2 weeks?<br>1.Yes 2.No (Skip to *H2*)	☐

H1a	What kind of medication has been taken: (multiple choice, try to write the name of the medication)<br>1.Hypolipidemic 2.Antihypertensive drugs 3.Oral hypoglycemic drugs 4.Insulin 5.Painkillers 6.Anticoagulants 7.Sleeping pills 8.Drugs for asthma 9.Diuretics 10.Antibiotics 11.Hormonal drugs 12.Thrombolytics 13.Aspirins 14.Contraceptive<br>15.Other : ________ (Remarks medication name: ______________)	□ □<br>□ □<br>□ □<br>□ □<br>________
H2	**Have you supplemented with any of the following health supplements in the last 6 months: (multiple responses allowed)**<br>1.Vitamins 2.Multivitamins 3.Fish oil 4.Lycopene 5.Selenium 6.Calcium 7.Zinc 8.Iron 9.Vitamin-mineral complex 10.Mineral complex 11.Folic acid<br>12.Other : _______ (Remarks health product name: ____________)<br>13.None	□ □<br>□ □<br>□ □<br>________

3.3 Menstrual and reproductive history (ask females only, males skip to L1)

Serial number	Description	Fill in the answer sheet
**J1 Menstrual history**		
J1a	Age of first menstruation	____Years
J1b	Duration of menstruation	____Day
J1c	Menstrual cycles	____Day
J1d	Have you been having regular periods?<br>1.Yes (If yes, skip to *J1g*) 2.No	□
J1e	When did your periods start to be irregular?	From ____ Year

		____Month
J1f	Symptoms of irregular menstruation? (Multiple choice)<br><br>1. Early menstruation 2. Delayed menstruation 3. Hypermenorrhea 4. Hypomenorrhea 5. Amenorrhea 6. Other_______	□__________
J1g	Do you have dysmenorrhea?<br>1. Yes 2. No (If no, skip to *J1i*)	□<br><br>__Year__Month
J1h	How often do you have menstrual cramps?<br><br>1. once a month 2. once every three months 3. once every six months 4. others _________	□<br><br>__Year__Month
J1i	Are you currently menopausal? 1. Yes 2. No	□ Age of menopause_ Years
K1 Reproductive history		
**K11**	**Do you have a reproductive history ?**<br>1. Yes 2. No (if no, skip to *L1*)	□
K1a	Age at first pregnancy	__________Years
K1b	Number of pregnancies and deliveries	Pregnancy:___times;<br><br>Delivery:___times
K1c	Number of existing children	Boy:____People ; Girl: People
K1d	Number of abortions	_________times
K1e	Number of preterm deliveries	_________times
K1f	Number of stillbirths	_________times
K1g	Number of abnormal fetuses	_________times

Part IV Professional History

<table>
<tr><th>Serial number</th><th colspan="6">Description</th><th>Fill in the answer sheet</th></tr>
<tr><td>L1</td><td colspan="6">Start of working time in the mine</td><td>________ Year_____Month</td></tr>
<tr><td>L2</td><td colspan="6">Current working status：1.Incumbency (skip to L4) 2.Transfer 3.Retirement 4.Other___</td><td>□________</td></tr>
<tr><td>L3</td><td colspan="6">Termination of working hours in the factory mine</td><td>________ Year_____Month</td></tr>
<tr><td rowspan="2">L4</td><td colspan="7">Work history at the factory and mine, fill in one line for each type of work. (Protective measures: 0.None 1.Mask 2.Face shields 3.Protective eyewear 4.Overalls 5.Helmet 6.Labor protection shoes 7.Glove 8.Other:_____)</td></tr>
<tr><td>Start time</td><td>End time</td><td>The name of the branch</td><td>Workshop name</td><td>Type of work</td><td>Stove number</td><td>Protective measures (multiple selections)</td></tr>
<tr><td>L4a
First</td><td>____ Year ____ Month</td><td>____ Year ___ Month</td><td></td><td></td><td></td><td></td><td>□□□□□□□
Other:________</td></tr>
<tr><td>L4b
Second</td><td>____ Year ____ Month</td><td>____ Year ___ Month</td><td></td><td></td><td></td><td></td><td>□□□□□□□
Other:________</td></tr>
<tr><td>L4c
Third</td><td>____ Year ____ Month</td><td>____ Year ___ Month</td><td></td><td></td><td></td><td></td><td>□□□□□□□
Other:________</td></tr>
<tr><td>L4d
Forth</td><td>____ Year</td><td>____ Year</td><td></td><td></td><td></td><td></td><td>□□□□□□□
Other:________</td></tr>
</table>

	___ Month	___ Month					
L4e Fifth	___ Year ___ Month	___ Year ___ Month					□□□□□□□ Other:________
L4f Sixth	___ Year ___ Month	___ Year ___ Month					□□□□□□□ Other:________
L4g Seventh	___ Year ___ Month	___ Year ___ Month					□□□□□□□ Other:________
L4h Eighth	___ Year ___ Month	___ Year ___ Month					□□□□□□□ Other:________

L5 Other job information

L5a	Change of uniforms for work: 1.none 2.occasionally 3.often 4.daily	□
L5b	Smoking in the workshop: 1.none 2.occasionally 3.often 4.daily	□
L5c	Eat in the workshop: 1.none 2.occasionally 3.often 4.daily	□
